# *In silico* structural and functional characterization of *Antheraea mylitta* cocoonase

**DOI:** 10.1186/s43141-022-00367-8

**Published:** 2022-07-11

**Authors:** Sneha Sneha, Dev Mani Pandey

**Affiliations:** Department of Bioengineering and Biotechnology, Birla Institute of Technology, Mesra, Ranchi, 835215 Jharkhand India

**Keywords:** *Antheraea mylitta*, Cocoonase, *In silico*, Silk degumming, *Antheraea pernyi*

## Abstract

**Background:**

Cocoonase is a serine protease present in sericigenous insects and majorly involved in dissolving of sericin protein allowing moth to escape. Cocoon structure is made up of sericin protein which holds fibroin filaments together. Cocoonase enzyme hydrolyzes sericin protein without harming the fibroin. However, until date, no detailed characterization of cocoonase enzyme and its presence in wild silk moth *Antheraea mylitta* has been carried out. Therefore, current study aimed for detailed characterization of amplified cocoonase enzyme, secondary and tertiary structure prediction, sequence and structural alignment, phylogenetic analysis, and computational validation. Several computational tools such as ProtParam, Iterative Threading Assembly Refinement (I-TASSER), PROCHECK, SAVES v6.0, TM-align, Molecular Evolutionary Genetics Analysis (MEGA) X, and Figtree were employed for characterization of cocoonase protein.

**Results:**

The present study elucidates about the isolation of RNA, cDNA preparation, PCR amplification, and *in silico* characterization of cocoonase from *Antheraea mylitta*. Here, total RNA was isolated from head region of *A. mylitta*, and gene-specific primers were designed using Primer3 followed by PCR-based amplification and sequencing. The newly constructed 377-bp length sequence of cocoonase was subjected to *in silico* characterization. *In silico* study of *A. mylitta* cocoonase showed 26% similarity to *A. pernyi* strain Qing-6 cocoonase using Blastp and belongs to member of chymotrypsin-like serine protease superfamily. From phylogenetic study, it was found that *A. mylitta* cocoonase sequence is closely related to *A. pernyi* cocoonase sequence.

**Conclusions:**

The present study revealed about the detailed *in silico* characterization of cocoonase gene and encoded protein obtained from *A. mylitta* head region. The results obtained infer the presence of cocoonase enzyme in the wild silkworm *A. mylitta* and can be used for cocoon degumming which will be a valuable and cost-effective strategy in silk industry.

**Supplementary Information:**

The online version contains supplementary material available at 10.1186/s43141-022-00367-8.

## Background

Among animal groups on the planet, insects are the most prosperous and are present in every corner of the world [1]. The advantage of insect’s adaptabilities is associated with their long-term evolution process into the environment, such as reproduction ability, short life cycle, and favorable small size to hide them. Additionally, insects enclose incisive life-cycle strategies, such as diapause [2], mimicry [3] and aposematic signals [4], and long-distance migration [5, 6], which are favorable for survival and population growth. Few holometabolous insects have adapted to cocoon formation as one of the effectual evolutionary strategies that helps to protect immobile pupa from mechanical damage, natural predators, parasites, and other adverse factors.

**Fig. 1:**
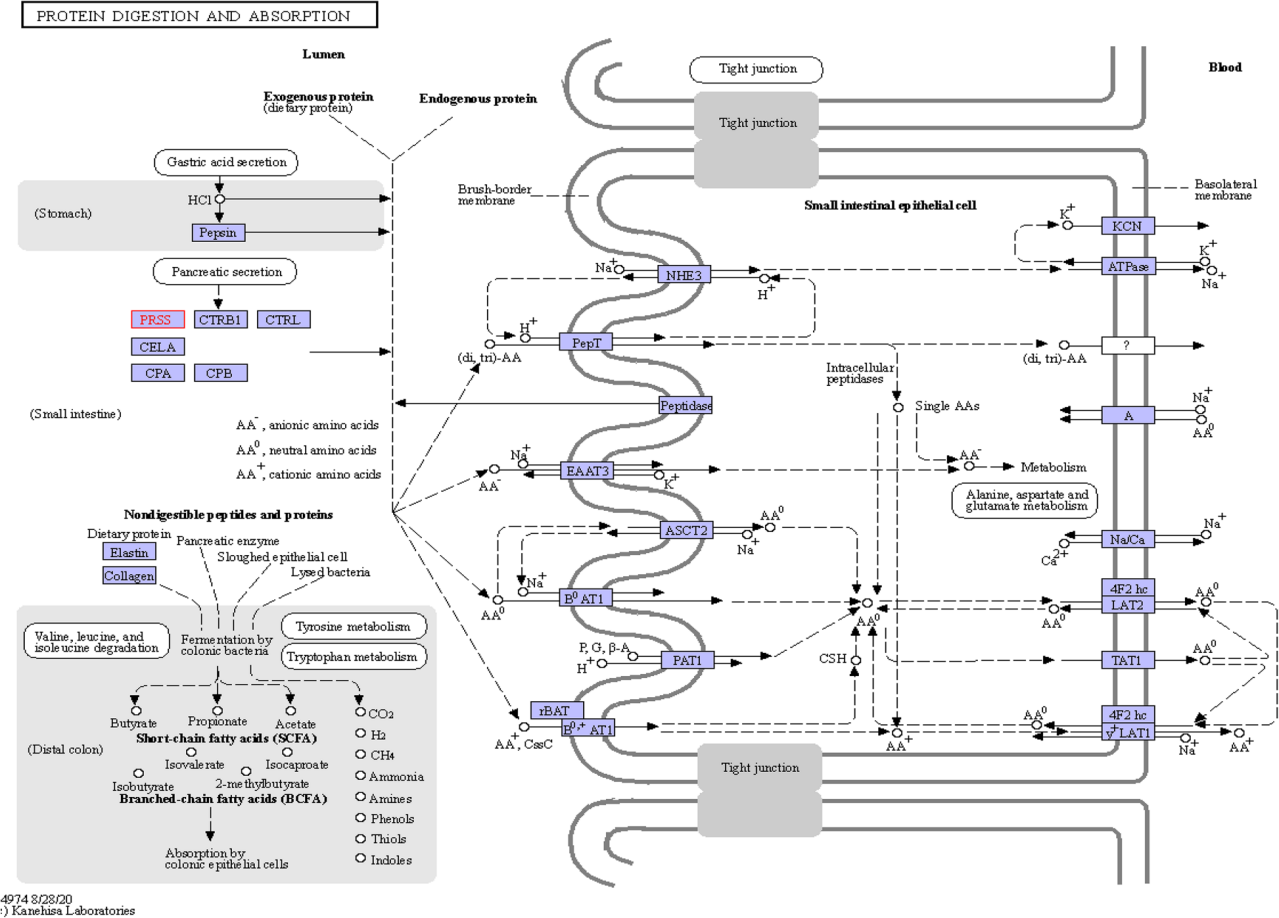
Metabolic pathway of trypsin (EC 3.4.21.4) (synonym of cocoonase) in protein digestion and absorption (KEGG database at http://www.genome.jp/kegg updated 28th Aug 2020)

Significant population of insects from Lepidoptera, Coleoptera, Hymenoptera, and Neuroptera [7–9] are capable of spinning. Mature insect larvae spun raw protein material (sericin and fibroin) secreted by its silk gland [10] to build cocoon, for instance, cocoon of domestic silk moth *Bombyx mori*, *Antheraea pernyi*, and *Antheraea mylitta* [11–13]. The report from previous study highlights the presence of a protease that hydrolyzes sericin, making the cocoon soft, and helps the moth to escape out [14, 15]. The metabolic pathway of peptide digestion is an important phenomenon of trypsin protease (gene name PRSS; https://www.genome.jp/entry/hsa:5644+hsa:5645+hsa:5646) and hydrolase enzyme (EC no. 3.4.21.4, www.brenda-enzymes.org) which are responsible for breakdown of peptides and related compounds (KEGG database at (http://www.genome.jp/kegg, Fig. 1). Cocoonase (synonym to trypsin, https://www.brenda-enzymes.org/enzyme.php?ecno=3.4.21.4#SYNONYM) is a naturally occurring enzyme that is functionally similar to trypsin. Cocoonase was first described in moths and is present as a single-copy gene [16]. However, recent work has identified multiple cocoonase duplication events in the *Heliconius melpomene* genome, resulting in at least five duplicates of recent origin [16].

Cocoonase enzyme is also well known as serine-trypsin protease or trypsin-like protease enzyme. Both enzymes are grouped in protease category and catalyze the breaking of peptide bonds and functionally defined with EC no. 3.4.21.4. The cocoonase gene coding sequence was unraveled gradually [17, 18], and its application in degumming has been also reported [19–25]. The boiling of cocoon in water dissolves sericin protein [26], and continuous raw silk filament is reeled and the whole process is known as silk degumming. Also, usage of chemical methods in Industrial Avenue for silk degumming of cocoons is commonly rampant. However, the usage of chemicals like soda, soap, detergents, alkaline, and alkali solution affects both sericin and fibroin, thus hampering the properties of tasar silk-like natural color, texture, and softness [27–29].

Therefore, it is expected that enzymatic cocoon degumming will be beneficial and may help to retain natural color, texture, and softness of tasar silk. Additionally, enzymatic methods have other advantages also as it is economical, eco-friendly, and biodegradable [30]. Hydrolyzing activity of cocoonase [31, 32] on sericin is similar as of trypsin. A study elucidating present and future perspective of cocoonase enzyme and its possible role in textile industry has been published [28]. Gene editing technique like CRISPR/Cas9-based *Bombyx mori* cocoonase gene editing has been the first experimental and phenotypic evidence showing that cocoonase is a cocoon breaking determining factor [33]. Using transcriptomic and genomic data heliconiine cocoonase gene expression across additional tissues, reconstructing their phylogenetic relationships, and examining the rates of gene duplication and deletion have already been described [34].

However, there is no detailed information available about cocoonase gene from *A. mylitta* silkworm. Furthermore, utilizing cocoonase-based degumming of cocoon strategy requires other information like ample production of cocoonase and its concentration-based degumming activity. Therefore, in present study, an effort has been made to find cocoonase gene and its characterization. Here, RNA was isolated from *A. mylitta* head region and gene amplification, and molecular study on the cocoonase gene from *A. mylitta* has been described. Furthermore, characterization of the coding nucleotide sequences predicting the tertiary and quaternary structure [35, 36] along with interaction of potential ligands focused on the active site residues of putative cocoonase protein of *A. mylitta (AmCoc)* has been done. The obtained findings infer the presence of cocoonase enzyme in the wild silkworm, *A. mylitta*. Gained information from present study can be utilized for the production of recombinant cocoonase and cocoon degumming.

## Methods

### Natural habitat of Antheraea mylitta and sample collection

**Fig. 2:**
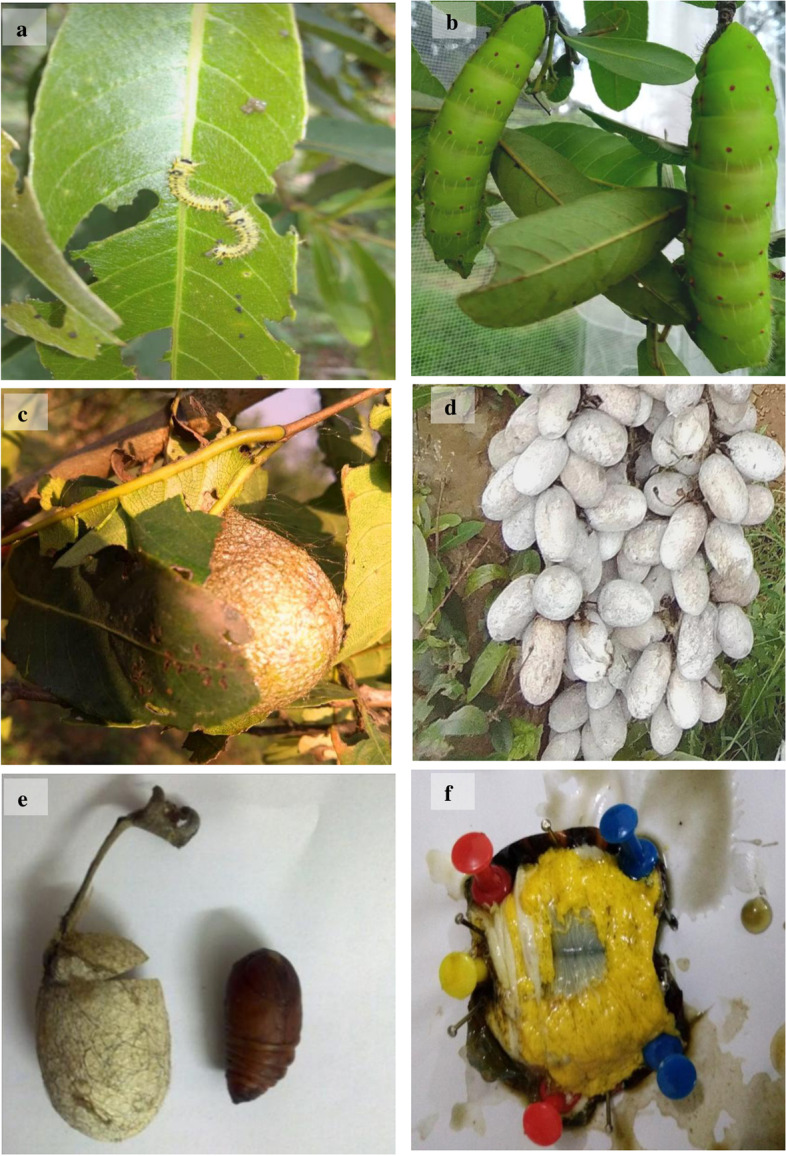
*Antheraea mylitta*. **a** 2nd instar larva stage feeding on *T. tomentosa*, **b** 4th larvae stage in natural habitat, **c** and **d** *Antheraea mylitta* cocoons on the *Terminalia tomentosa* in natural environment, **e** 5th instar pupa sample, and **f** dissection of 5.th instar pupa sample for RNA isolation

*Antheraea mylitta* Drury, tasar silkworm, is a wild sericigenous, polyphagous insect spread in different geographical zones in India [37]. Tasar silkworm late pupa (Fig. 2 a–b) and cocoon samples (Fig. 2 c–d) of *A. mylitta* Drury, feed on *Terminalia tomentosa* and *Shorea robusta* [38], were collected from natural habitat of Central Tasar Research and Training Institute, Ranchi, India. Fifth larval stage is the perfect stage to produce cocoonase in maximum. The *A. mylita* Drury cocoon research samples were kindly provided by Dr. J. P. Pandey (scientist D, CTR &TI, Ranchi, India). CTR&TI is the flagship research institute catering to the R&D need of tropical and temperate (oak) tasar sectors. Late pupal stage samples of 125 days old were selected for RNA isolation from brain tissues (Fig. 2 e–f) via TRIzol® extraction protocol [39]. The *Antheraea mylitta* pupa samples were disinfected using 70% ethanol and dissected under sterilized condition. Dissected pupa head (anterior portion) was subjected for RNA isolation.

### Retrieval of cocoonase gene sequence and primer designing

The hydrolysis of sericin protein is catalyzed by cocoonase enzyme; therefore, in NCBI database, cocoonase entry was searched, and its sequence from *Antheraea pernyi* strain was retrieved (NCBI accession no. gi|295,682,679|). The above sequence was submitted to tblastn for getting its coding sequence (GenBank: ADG26770.1). Four sets of primers (including forward and reverse) were obtained using online primer designing tool (Primer3) with optimized parameters such as GC%, length of primer, and amplicon size. List of primer sets used in PCR amplification has been shown in Table 1, as *A. mylita* and *A. pernyi* wild silk moth belongs to the same genus. Therefore, cocoonase protein sequence (ADG26770.1) was selected as template for primer designing.

**Table 1:** Primer sets procured from Xcelris, India

Name	Primers	Annealing temp. (°C)	Amplicon size (bp)
ApCoc1	TACTATTGGCTTGTGCCATTTTT (Forward)	49.6	769
	ATATACACCAGGGTTTCCAGGAC (Reverse)		
ApCoc2	AGTCAAAGAACGAATGATGTTGGG (Forward)	50.5	686
	CGGAGTGCTGTGACATTTGC (Reverse)		
ApCoc3	ACTATTGGCTTGTGCCATTTTT (Forward)	48.5	746
	GTATCCAACCACGGAGTGTC (Reverse)		
ApCoc4	TTTACTATTGGCTTGTGCCATTT (Forward)	45.8	771
	TTTTAAACTCCTGCAGTCTTTCG (Reverse)		

### PCR amplification and sequencing

Four sets of gene-specific primers were used for PCR amplification following the PCR preparations of TaKaRa™. PCR amplification was performed in a final volume of 12.5 μL containing cDNA (150 ng), 10 pmol of the each primers, mixture of dNTPs (Sigma) having concentration of 250 μM, 10 × Taq Polymerase Buffer, and 0.625 U of Taq DNA polymerase (TaKaRa™). The reaction conditions for PCR set up were as follows: an initial denaturation step at 95 °C for 1 min, 35 amplification cycles of denaturation at 95 °C for 30 s, annealing at 49 °C for 30 s, and primer extension at 72 °C for 90 s, followed by a final extension at 72 °C for 10 min with TaKaRa PCR thermal cycler Dice (Thermo Fisher Scientific, USA). The primer set which gave single band amplification with cDNA was selected, and amplified PCR product was submitted for sequencing to Chromous Biotech, Bangalore, India.

### Physicochemical characterization

Primary sequence analysis was performed by calculating the physicochemical properties of retrieved protein sequences which include isoelectric point (pI), molecular weight (MW), instability index (II), aliphatic index (AI), and GRAVY or grand average of hydropathicities by using ExPASY-ProtParam tool (http://web.expasy.org/protparam/) [40]. The secondary structural features (like helix, turn, sheet, coil, etc.) were predicted by SOPMA (http://npsa-pbil.ibcp.fr/cgi-bin/npsa_automat.pl?page=/NPSA/npsa_sopma.html) [41] and CFSSP: Chou and Fasman Secondary Structure Prediction server (http://cho-fas.sourceforge.net/), [42, 43]. The PredSL (http://aias.biol.uoa.gr/PredSL/) [44] and PredictProtein (https://predictprotein.org/) [45] were used to predict subcellular location of the derived target protein. Protein dynamics information is also important for understanding protein function. DynaMine web server quickly produces profile describing statistical potential for fast backbone protein movements directly from amino acid sequence available at http://dynamine.ibsquare.be/ [46].

### Modeling and structural and functional analysis

3D protein structure of AmCoc was determined by QUARK and I-TASSER server, https://zhanglab.dcmb.med.umich.edu/I-TASSER/ [47, 48]. The stereo-chemical quality assessment of predicted protein structure was performed by PROCHEK [49–52], RAMPAGE [47, 53, 54], and UCLA-DOE LAB SAVES server (http://services.mbi.ucla.edu/SAVES/). Potential deviations and structural alignment were calculated with TM-align web server [55] (https://zhanggroup.org/TM-align/) for root-mean-square deviation (RMSD). The potential errors were checked in predicted tertiary protein model, while z-score value was calculated and compared with target template by ProSA-web tool [56] (https://prosa.services.came.sbg.ac.at/prosa.php). This displays overall quality and if the input structure lies within the score range for the native proteins of similar size [24, 57].

### Sequence annotation and NCBI submission

PCR amplified and obtained cocoonase sequence was analyzed by various computational and web-based online tools. DNA TIS Miner tool [58] (available at http://dnafsminer.bic.nus.edu.sg/) was used for finding start codons and ORF finder tool (http://www.bioinformatics.org/sms2/orf_find.html) for determining ORFs in the cocoonase sequence. The number of exons, exon position, and exon was predicted by GeneWise tool. Conserved domain tool available at https://www.ncbi.nlm.nih.gov/Structure/cdd/cdd.shtml [59–61] reports the functional motifs [62] location and was used to predict the presence of conserve domain in predicted protein model of cocoonase (KM388539.1). Moreover, protein domain and domain architecture were analyzed with SMART tool [63] (http://smart.embl-heidelberg.de/), and the presence of motif was performed using MEME tool (https://meme-suite.org/meme/tools/meme). Structural Classification of Proteins (SCOP) available at http://scop.mrc-lmb.cam.ac.uk/scop/ provides comprehensive structural and evolutionary relationships between all proteins whose structure is known [64, 65].

### BLAST against Antheraea mylitta genome

Obtained cocoonase sequence (KM388539.1) was subjected to NCBI blast (https://www.ncbi.nlm.nih.gov/) against *A. mylitta* GenBank assembly GCA_014332785.1 (AM_v1.0).

## Results

**Fig. 3:**
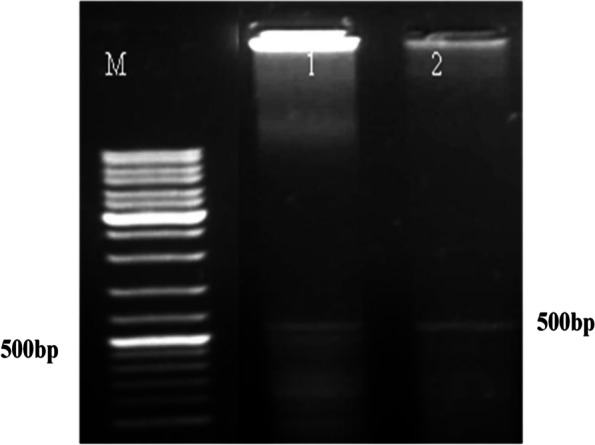
M, marker, DNA samples amplified with ApCoc4 primer in replicates (amplicon size ~ 500 bp) in L1 and L2

**Fig. 4:**
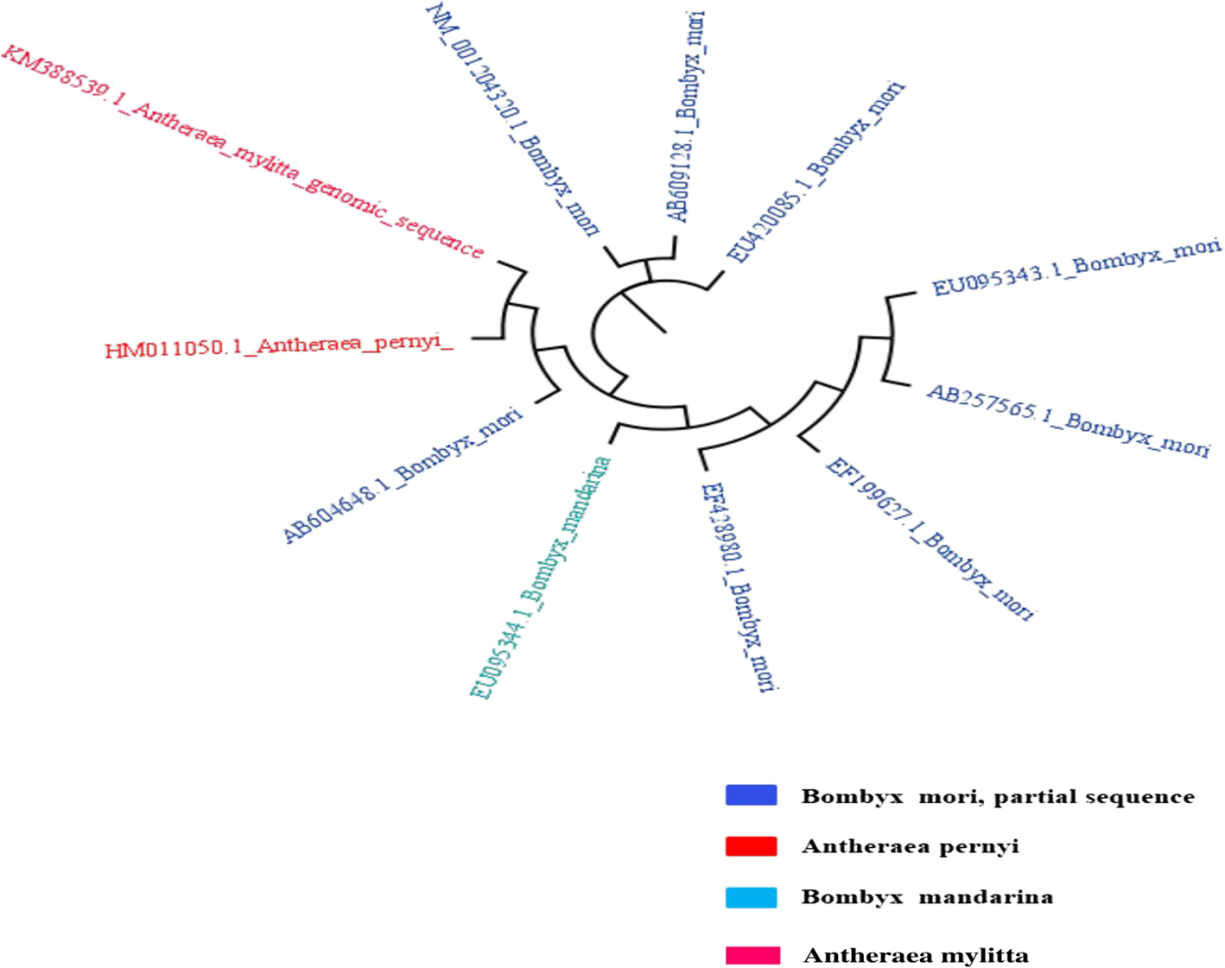
Phylogenetic tree constructed with AmCoc (accession no.KM388539.1) and enlisted nucleotide cocoonase sequences from homologues

Details of silkworm late pupa (Fig. 2 a–b), cocoon samples (Fig. 2 c–d), fifth instar larva of *A. mylitta* (Fig. 2e) moth, and sampling of brain tissues for RNA isolation (Fig. 2f) have been depicted. PCR amplification with gene-specific primer and optimization in respect to annealing temperature, number of cycles, and concentration of the template DNA was performed. The PCR thermal profile cycle was maintained as follows: 95 °C, 1 min; 95 °C, 30 s; 49 °C, 30 s; 72 °C, 90 s; and 72 °C, 10 min for 35 cycles with ApCoc4 primer set. Amplified PCR product (amplicon size ~ 500 bp) of *A. mylitta* with primer ApCoc4 was submitted for sequencing (Fig. 3). Obtained nucleotide sequences of *A. mylitta* were subsequently analyzed, assembled, and annotated. Following sequence assembly, a new sequence of AmCoc (377 bp) was constructed. Newly constructed *AmCoc* nucleotide sequence was checked for the similarity using BLAST with *A. pernyi* cocoonase gene reported in NCBI database (ADG267710.1) and was found to be identical (query coverage — 11%; maximum identity 97%). The predicted gene constitutes 1 exon with 48% GC content having 122 amino acids in translated protein sequence as explained by GeneWise algorithm (Table 2). DNA TIS Miner tool-based analysis for finding translation initiation sites (TIS) total 4 positions was found. But as per ORF finding tool, at nucleotide position 253, it can be confirmed that gene may start with an open reading frame, and ORF is shown in red-colored font (Table 3). Phylogenetic tree was constructed with new sequence with GenBank ID > gi|731,516,038|gb|KM388539.1| UNVERIFIED: *Antheraea mylitta* genomic KM388539.1 (Table 4), showed that it is closely related to *A. pernyi* strain qing_6 cocoonase-like protein mRNA sequence (GenBank ID HM011050.1, Fig. 4). Cooconase gene NCBI blast result shows that sequences are matched with *A. mylitta* isolate AMDABA2020 scaffold18_size7685921 and whole genome shotgun sequence (Supplementary Fig. S1) showing only 2 matches with *A. mylitta* isolate AMDABA2020 scaffold18_size7685921 (Supplementary Fig. S2). Smith et al. [16] has reported that cocoonase gene is a single-copy gene in several butterfly and moth genomes (the silk moth *Bombyx mori*, diamond backed moth *Plutella xylostella* and monarch butterfly *Danaus plexippus*, and the *Glanville fritillary* (*Melitaea cinxia*). It needs to mention that cocoonase protease activity might be comparable with trypsin protease enzyme activity, because both the abovementioned proteases are enrolled with identical Enzyme Commission number (EC 3.4.21.4), and also, trypsin is synonym to cocoonase.

**Table 2:** Prediction of exon, exon position, exon range, exon length, and GC content by GeneWise tool

Gene	Exon	Strand	Exon type	Exon range	Exon length	GC content
1	1	+	Initial	1–375	375	48%

**Table 3:** Prediction of start codon, score, position, and Kozak consensus sequence by DNA TIS miner

No. of ATG’s from 5′ end?	Score	Position (bp)	Kozak consensus [AG]XXATGG
4	0.755	253	AXXATGC
3	0.664	55	GXXATGA
2	0.506	188	GXXATGC
1	0.356	58	GXXATGC

**Table 4:** *Antheraea mylitta* cocoonase (AmCoc) nucleotide sequence deposited in NCBI

** > KM388539.1 UNVERIFIED: *Antheraea mylitta* genomic sequence**
TCTATTGGCTTGTGCCATTTTTTTCTATTGGCTTGTGCCATTTTTAACAGCAGATGATGCTCGTATTTCGCGCCAGTTTTTCAAGCCCGGCGGAAAAGGGCCGGGGAACAGGGGACCGAATTTTTCCACCGCACGGTCAGTAAAAGGCCGACGGAAAAATTTCACTTCCACCACCACCGCATCGATGCGCCCCTTGAGATAAAAAAAAAAAGGCATCTTGCCGGGGCCTAACTTATCAATAGAAGGAAAATGCTTTGCCGAATTTTTCCGCAAAACAGAAAAAACCCCGGGGCATATATTCCCGGCAACCGGCAAATCCCCAAGTACCCAAAAAACGACGGCGAATTCCGGCCACCACCCCGGGGTTGGACAAA

**Fig. 5:**
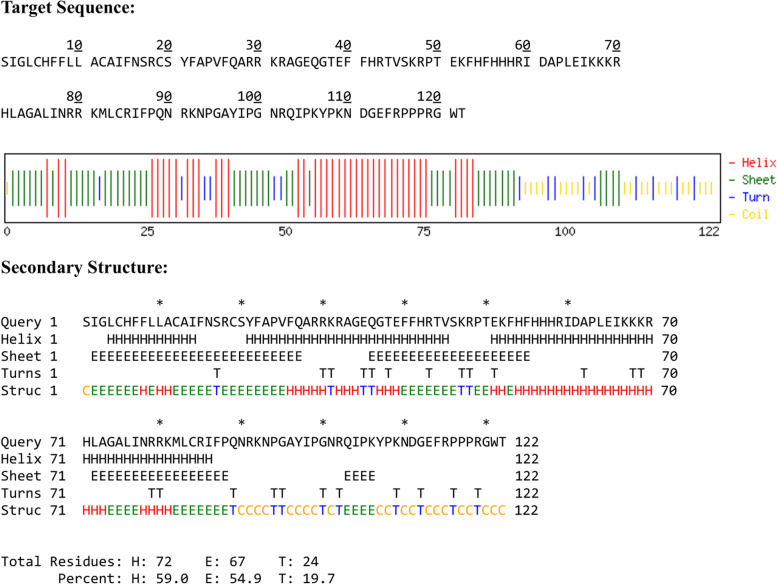
Secondary structure of AmCoc

**Fig. 6:**
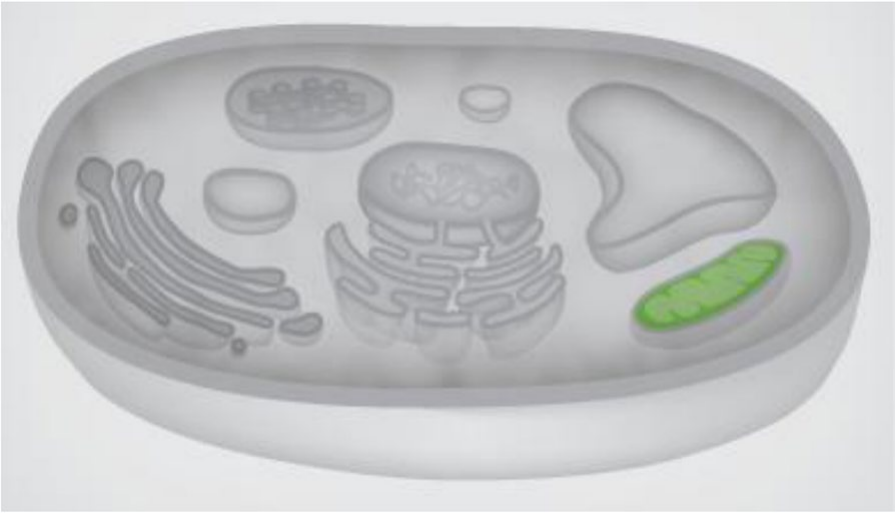
Subcellular localization of AmCoc mitochondrial protein predicted by PredictProtein tool

**Fig. 7:**
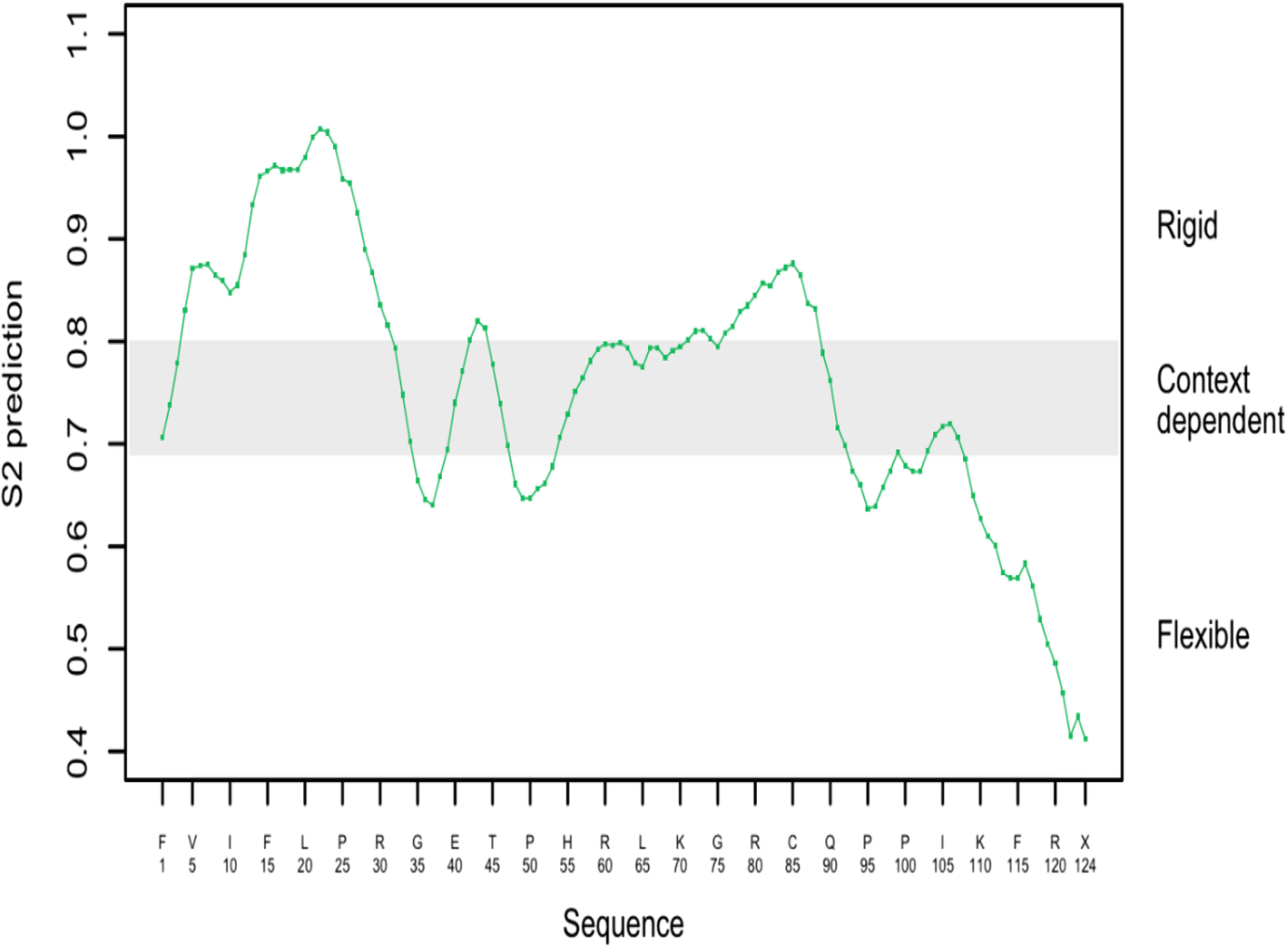
Prediction of dynamic nature of AmCoc protein using DynaMine server: our results showed that the most of regions of AmCoc are rigid, and there are only nine flexible regions with the lowest predicted S2 value which are Phe115 (0.57), Arg116 (0.58, Pro117 (0.56), Pro118 (0.53), Pro119 (0.51), Gly121 (0.46), Trp122 (0.41), and Thr123 (0.43)

AmCoc physicochemical parameters were derived using ProtParam tool (Table 5) that corresponds with 124 amino acid residues, molecular weight of 14.681 kDa, and computed pI of 10.97. The deduced amino acid sequence contains 7 negatively charged (− R, Asp + Glu) and 25 positively charged (+ R, Arg + Lys) amino acid residues. The value of instability index, aliphatic index, and grand average of hydropathicity (GRAVY) was 53.44, 59.59, and − 0.733, respectively. The highest frequency of amino acids in the sequence is arginine (12.3%), alanine (10.3%), followed by proline (9.5%). The secondary structure prediction of AmCoc sequence is shown in Fig. 5 and Supplementary Fig. S3. Helix, sheet, and turn (59%, 54.9%, and 19.7%, respectively) as secondary structures were predicted by Chou–Fasman web server. Subcellular location of the derived protein was determined by the PredictProtein (Fig. 6), while using PredSL tools, it was observed that it is a mitochondrial protein. The stability of the derived amino acid sequence was determined by DynaMine web server exhibiting that maximum amino acid residues lay in the rigid area (Fig. 7).

**Table 5:** Physicochemical parameter of cocoonase computed by ProtParam tool

Physicochemical properties	Cocoonase (KM KM388539.1; *A. mylitta*)
Number of amino acid residues	124
Theoretical pI	10.97
Instability index	53.44
Aliphatic index	59.59
Grand average of hydropathicity (GRAVY)	− 0.733
− R (Asp + Glu)	7
+ R (Arg + Lys)	25

**Fig. 8:**
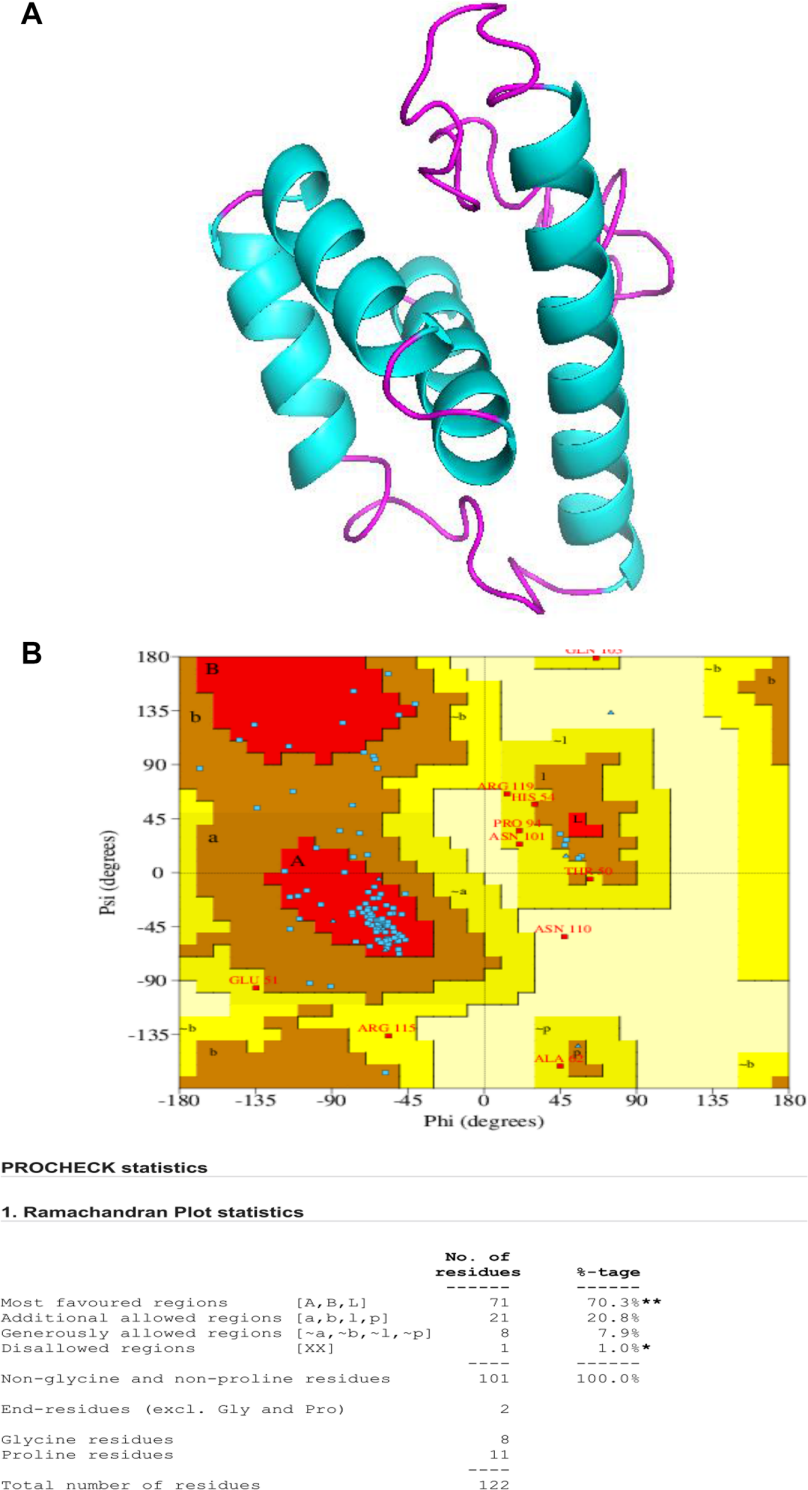
Predicted 3D model (I-TASSER) of **a** AmCoc encoded protein where α-helices are shown in cyan color and coils are in magenta. **b** Ramachandran plot (between φ-ψ torsion angles) of the predicted protein is shown where the cream areas correspond to sterically disallowed regions except glycine, red and brown areas correspond to sterically allowed regions for alpha-helical and beta-sheet conformations, and yellow areas correspond to allowed regions for the left-handed alpha-helix (A right-handed alpha-helix, B beta-sheet, and L left-handed alpha helix)

**Fig. 9:**
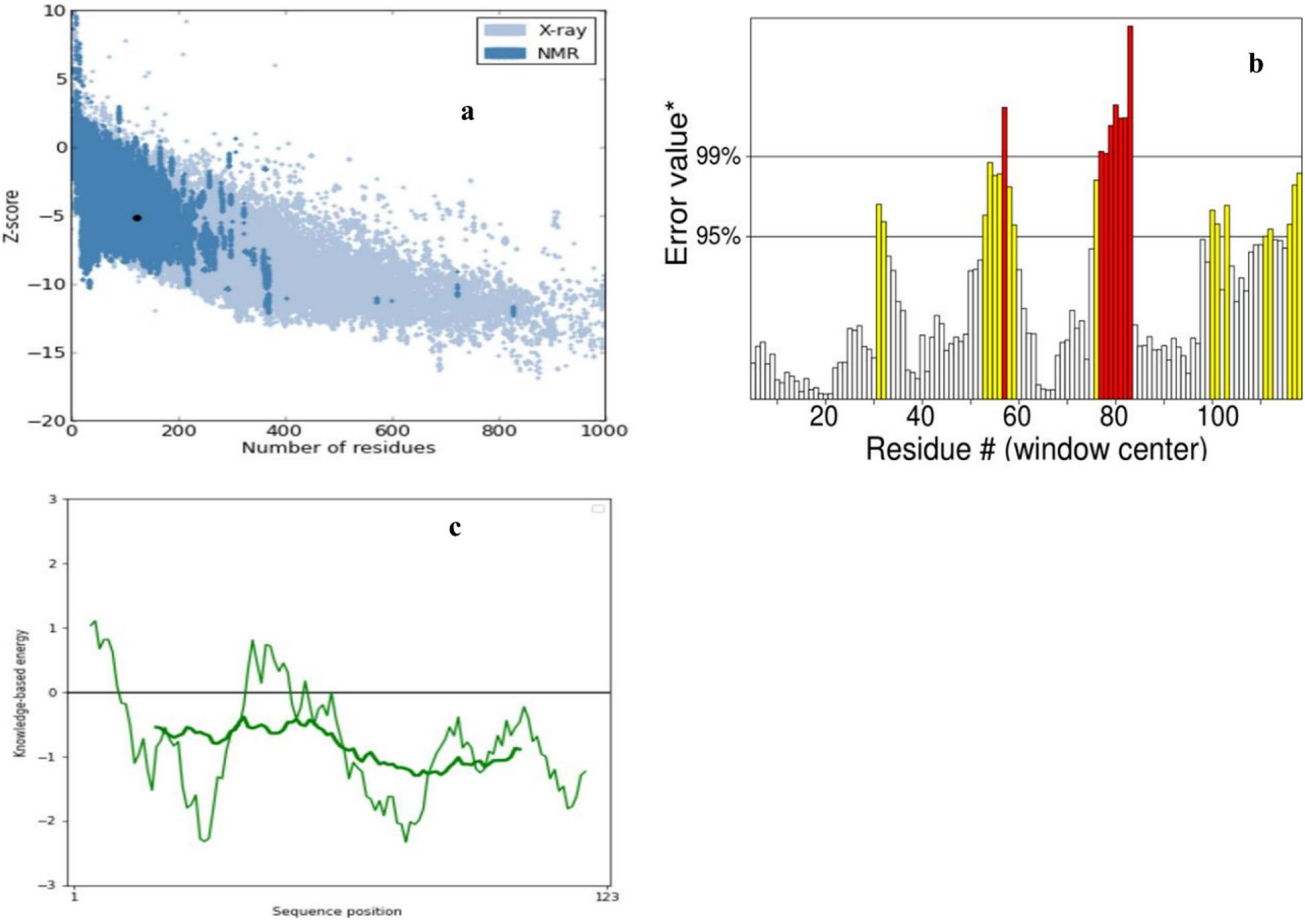
(**a**) ProSA-web z-scores of all protein chains in PDB determined by X-ray crystallography (light blue) or NMR spectroscopy (dark blue) with respect to amino acid chain length. The z-scores of AmCoc is highlighted as large dots Z-score for template protein structure–AmCoc -4.92; (**b**) SAVES ERRAT graph of AmCoc; (**c**) Energy plot of AmCoc

**Fig. 10:**
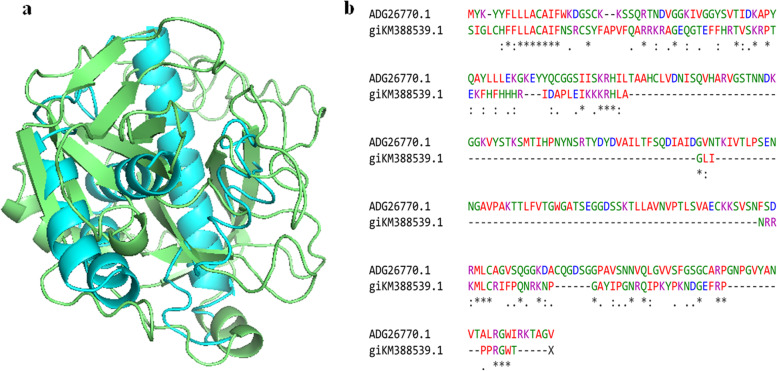
**a** Superimposed three-dimensional structure model of template ApCoc (red) and target AmCoc (blue). **b** Pairwise alignment for ApCoc protein sequence and ApCoc protein sequence with ClustalW

**Fig. 11:**
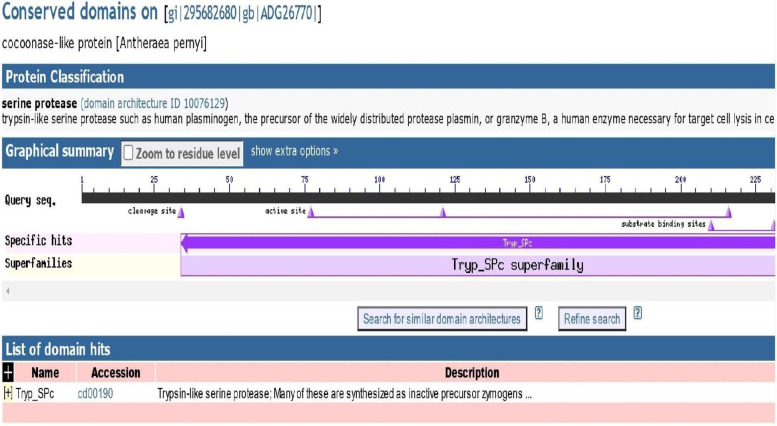
The conserve domain identification of cocoonase by NCBI Conserved Domain Database

The 3D model of *A. mylitta* cocoonase protein (KM388539.1) was predicted by QUARK and I-TASSER servers and viewed by PyMol (Fig. 8a). Helices and loops were colored in cyan and magenta, respectively. The best predicted protein structure was selected based on TM score (0.3461). Furthermore, structural validation and quality assessment of the model were carried out using various tools such as PROCHEK, RMSD, RAMPAGE, and z-score. Ramachandran plot-based analysis showed that 70.3% of residues were in the most favored region, 19.7% in the allowed region, while 2% in the disallowed region (Fig. 8b). Also, SAVES ERRAT (78%) and z-score for the AmCoc-predicted protein structure was found to be − 4.92 (Fig. 9). The structural alignment was performed with TM-align tool between AmCoc-predicted protein structure and ApCoc-predicted structure showing the *RMSD* = 5.68A and viewed in PyMol (Fig. 10). Moreover, protein domain and domain architecture were analyzed with SMART tool (http://smart.embl-heidelberg.de/, 41), and translated AmCoc protein belonged to a distinct SCOP superfamily d1kypa. The deduced amino acid of *A. mylitta* cocoonase sequence comprised of two motifs which are determined by MEME tool suite (Supplementary Fig. S4). Obtained results indicated that conserved domains of deduced amino acid sequence of cocoonase (Fig. 11) were a trypsin-like serine protease having active site from 75 to 200 query sequence and substrate binding site from 210 to 225 query sequences in NCBI. Both the motifs belong to trypsin-like serine protease, and cocoonase-like protein has been inferred as a conserved domain (cd00190) at the positions of 56–76 and 84–104 and each of 20 amino acid in length. Detailed comparative modeling and protein structure analysis have been performed to infer functional (and perhaps adaptive) differences of heliconiine cocoonase compared with the single-copy moth cocoonase [34].

## Discussion

Cocoonase is a very important protease enzyme responsible for hydrolyzing sericin of silk cocoon. A study using bioinformatics tools has been published showing that cocoonase is specific to Lepidoptera, and also, it existed before the occurrence of lepidopteran insects spinning cocoons [33]. The primary structure of cocoonase revealed about amino acid sequence arrangement, while secondary and tertiary structure of the protein illustrates the enzymatic function in-depth. The first attempt of the present study was to characterize a novel cocoonase gene amplified from cDNA of *A. mylitta* brain tissues using computational approaches. PCR amplification was obtained with primer set ApCoc4 (Table 1), and obtained amplified product was subsequently sequenced (Fig. 3). Sequence alignment [66, 67], phylogenetic analysis, motif identification, functional annotation, and structure analysis by homology modeling [68], elucidated that AmCoc shows similarity to proteases from other sericigenous insects such as *A. pernyi* and *B. mori*. The annotation of the newly constructed sequence AmCoc (377 bp) was used to search the presence of the serine protease domain, cd00190, using SMART tool. Also, modeling-based data of 30 individual cocoonases indicated that all the cocoonase enzymes have trypsin-like specificity, and also, significant differences were noticed among the surface residues of different cocoonase types which suggest that cocoonase enzyme shows varying adaptation to different chemical environments [34].

Finding ORF and translation initiation sites is important for understanding their key role and predicting the coding region in newly constructed sequence. Gene prediction was performed with GeneWise tool which shows exon positions, exon range, and length to a genomic DNA sequence [69] and listed in Table 2. Relatedness and distinction among linked genetic sequences have been explained by sequence alignment and represented pictorially in phylogenetic tree, defining an evolutionary descent of distinct species, organisms, or genus from a common ancestor [70, 71]. In the current study, phylogenetic analysis revealed that the obtained cocoonase sequence from *A. mylitta* (accession no. KM388539.1) belongs to the same clade of *A. pernyi* (ADG267710.1) and evolutionary related as shown in Fig. 4. PredictProtein and PredSL analysis showed that the target protein, *A. mylitta* cocoonase enzyme from head portion, is mitochondrial protein and possesses signal peptide [72, 73] (Fig. 6).

I-TASSER hierarchical protocol was used for automated protein structure prediction and structure-based function annotation that predicts and infers the secondary and tertiary structures, structural and functional annotations, ligand-binding sites, active sites, enzyme commission, and gene ontology terms [65]. The scale of accuracy for the predictions is based on confidence score (C-score) of the protein model, TM score (scale for measuring the structural similarity between two protein structures), and RMSD value (average distance of all residue pairs) [47, 74] as shown in Fig. 8. The structural alignment was performed between *A. mylitta* cocoonase predicted structure and *A. pernyi* cocoonase protein structure showing RMSD value = 5.68A and viewed in PyMol (Fig. 10). The RMSD superimposition value indicated that there is similarity among the target (AmCoc) and the template structure (ApCoc). *A. mylitta* cocoonase close structural similarity with the template cocoonase from *A. pernyi* (Fig. 10) suggests that there is a functional similarity with cocoonase from *A. mylitta* (RMSD = 5.68A, viewed in PyMol) 

 Cocoonase gene isolated from head region, characterization and its analysis in silk degumming have not been reported in *Antheraea* sp.; however, there are previous reports about the presence of cocoonase enzyme in *B. mori* silk moth (domestic) and *A. pernyi* (wild) and its role in silk degumming. Through *in silico* predictions, *AmCoc*-derived cocoonase gene sequence showed similarity with template sequence, and the presence of conserved domain and motif has been observed which belongs to trypsin-specific family (Fig. 11). Prediction of protein functions using 3D structure information, enzyme commission number, and ligand binding sites has been described using COFACTOR [75]. COFACTOR tool-based analysis of cocoonase protein predicted a template of PDB ID: 3cskA with EC number 3.4.14.4 (dipeptidyl-peptidase III belonging to hydrolase) and active site residues [6, 15, 43, 53, 54, 76]. Similar type prediction has also been described using *B. mori *cocoonase sequence [65]. The functional difference of enzyme isoforms was calculated using DEEPre tool based on enzyme EC number prediction by deep learning method (Supplementary Fig. S5). Domain and motif identification in protein is a vital step for better understanding of structural and functional inference of predicted protein [18, 77].

A detailed study on the genetic analysis of Indian tasar silk moth (*A. mylitta*) populations has been published [78]. However, no detailed information is available for *A. mylitta* cocoonase gene. Furthermore, the study about the cocoonase gene structure, copy number, chromosome location and its expression patterns, etc. in *A. mylitta* is of great significance. Here, cocoonase gene sequence was subjected to NCBI blast against GenBank assembly *Antheraea mylitta***—**GCA_014332785.1 (AM_v1.0) indicated the matching of sequences with *A. mylitta* isolate AMDABA2020 scaffold18_size7685921 whole genome shotgun sequence (Supplementary Fig. S1) having 2 matches only (Supplementary Fig. S2), although six copies of *cocoonase* has been reported in *Heliconius melpomene* and copy number varies across *H. melpomene* subpopulation [16]. Also, a detailed list about the copy number variation in cocoonase genes across 18 individuals of four *Heliconius melpomene* (Hm) subspecies has been elaborated [34]. Nowadays, the gene editing technologies are also being used to unravel the functionality of various genes. Recently, gene editing technique like CRISPR/Cas9 has been used to knock out cocoonase in the silkworm *B. mori *[33]. Detailed cocoonase gene expression analysis has not been performed in the present study, although PCR-based cocoonase gene amplification was seen in brain tissue only (Fig. 3). Detailed study about mRNA expression levels of cocoonases across multiple *H. melpomene* tissues (like mouth parts, antennae, head, and legs) has been described where high expression levels were indicative of an important function for cocoonase 3 and cocoonase 4 in the mouth part tissues [34].

## Conclusion

In summary, the present study describes about the isolation of RNA, cDNA preparation, PCR-based amplification, sequencing, and identification of cocoonase gene from head region of *A. mylitta*. Annotation resulted to the newly constructed cocoonase (*AmCoc*) sequence of 377 bp only. Phylogenetic analysis of ApCoc and AmCoc revealed their evolutionary relationship between different species. NCBI blast against GenBank assembly *Antheraea mylitta*—GCA_014332785.1 (AM_v1.0) indicated the matching of sequences with *A. mylitta* isolate AMDABA2020 scaffold18_size7685921 whole genome shotgun sequence. Secondary structure as well as 3D structure prediction of AmCoc cocoonase disclosed the detailed atomic structure, while I-TASSER predicted the most stable structure. AmCoc proteins were searched in PDB for predicting their structural closeness to the target in the PDB (3cskA) and active sites [6, 15, 43, 53, 54, 76]. EC predictions revealed that AmCoc cocoonase (dipeptidyl-peptidase III belonging to hydrolase) has EC number 3.4.14.4. Furthermore, AmCoc enzyme is a mitochondrial protein, which possesses signal peptide and serine protease domain. The present study broadens our knowledge about *A. mylitta* cocoonase (AmCoc) characteristics which may be helpful in further elucidating its full gene sequences and encoding protein. Obtained findings may further be utilized to add economical value of silk by altering the degumming process of cocoon and thereby retaining the texture and color of silk.

## Supplementary Information

**Additional file 1:** **Figure S1.** NCBI blast result of cocoonase sequence against Antheraea mylitta—GCA_014332785.1 (AM_v1.0). The blast result show that sequences are matched with *A. mylitta* isolate AMDABA2020 scaffold18_size7685921, whole genome shotgun sequence. **Figure S2.** NCBI blast result of cocoonase sequence against Antheraea mylitta—GCA_014332785.1 (AM_v1.0). The blast result indicated the 2 matches only with *A. mylitta* isolate AMDABA2020 scaffold18_size7685921, whole genome shotgun sequence. **Figure S3.** Secondary structure prediction of *Antheraea mylitta* cocoonase (AmCoc) from PSPIRED server: (a) Predicted helix, strand and coil of the protein (b) Secondary structure map of cocoonase. **Figure S4.** MEME tool based result of *Antheraea mylitta* cocoonase (AmCoc) of KM388539.1 showing two strong motifs in the sequence highlighted in red (MFCAGPPEGGKDSCQGDSGGP) at position 84–104 and in lime green (INKVPYQAYLLLQKBNEYFQC) at position 56- 76. **Figure S5.** Enzyme Commission numbers and active sites for *Antheraea mylitta* predicted cocoonase based on the template of PDB ID: 3cskA having C-score of 0.065. The predicted active-site residues are 9, 12, 25, 38, 42 and 77 is highlighted with magenta color code.

## Data Availability

Not applicable.

## Abbreviations

EC no: Enzyme Commission number
AmCoc: *Antheraea mylitta* Cocoonase
CTR&TI: Central Tasar Research and Training Institute
NCBI: National Center for Biotechnology Information search database
JTT model: Jones-Taylor-Thornton model
PCR: Polymerase chain reaction
ApCoc: *Antheraea pernyi* Cocoonase
SMART: Simple Modular Architecture Research Tool
SOPMA: Secondary structure prediction method
RMSD: Root-mean-square deviation
CDD: Conserved Domain Database
DNA TIS: DNA translation initiation site
GO: Gene ontology
ORF: Open reading frame
