# Supplementary material for: In silico structural and functional characterization of Antheraea mylitta cocoonase
Source: J Genet Eng Biotechnol. 2022 Jul 11;20:102. doi: 10.1186/s43141-022-00367-8 (PMC9273796; doi:10.1186/s43141-022-00367-8)
Supplement: Supplementary file 1 — Additional file 1: Figure S1. NCBI blast result of cocoonase sequence against Antheraea mylitta—GCA_014332785.1 (AM_v1.0). The blast result show that sequences are matched with A. mylitta isolate AMDABA2020 scaffold18_size7685921, whole genome shotgun sequence. Figure S2. NCBI blast result of cocoonase sequence against Antheraea mylitta—GCA_014332785.1 (AM_v1.0). The blast result indicated the 2 matches only with A. mylitta isolate AMDABA2020 scaffold18_size7685921, whole genome shotgun sequence. Figure S3. Secondary structure prediction of Antheraea mylitta cocoonase (AmCoc) from PSPIRED server: (a) Predicted helix, strand and coil of the protein (b) Secondary structure map of cocoonase. Figure S4. MEME tool based result of Antheraea mylitta cocoonase (AmCoc) of KM388539.1 showing two strong motifs in the sequence highlighted in red (MFCAGPPEGGKDSCQGDSGGP) at position 84–104 and in lime green (INKVPYQAYLLLQKBNEYFQC) at position 56- 76. Figure S5. Enzyme Commission numbers and active sites for Antheraea mylitta predicted cocoonase based on the template of PDB ID: 3cskA having C-score of 0.065. The predicted active-site residues are 9, 12, 25, 38, 42 and 77 is highlighted with magenta color code. [file 43141_2022_367_MOESM1_ESM.doc]

**Supplementary materials**


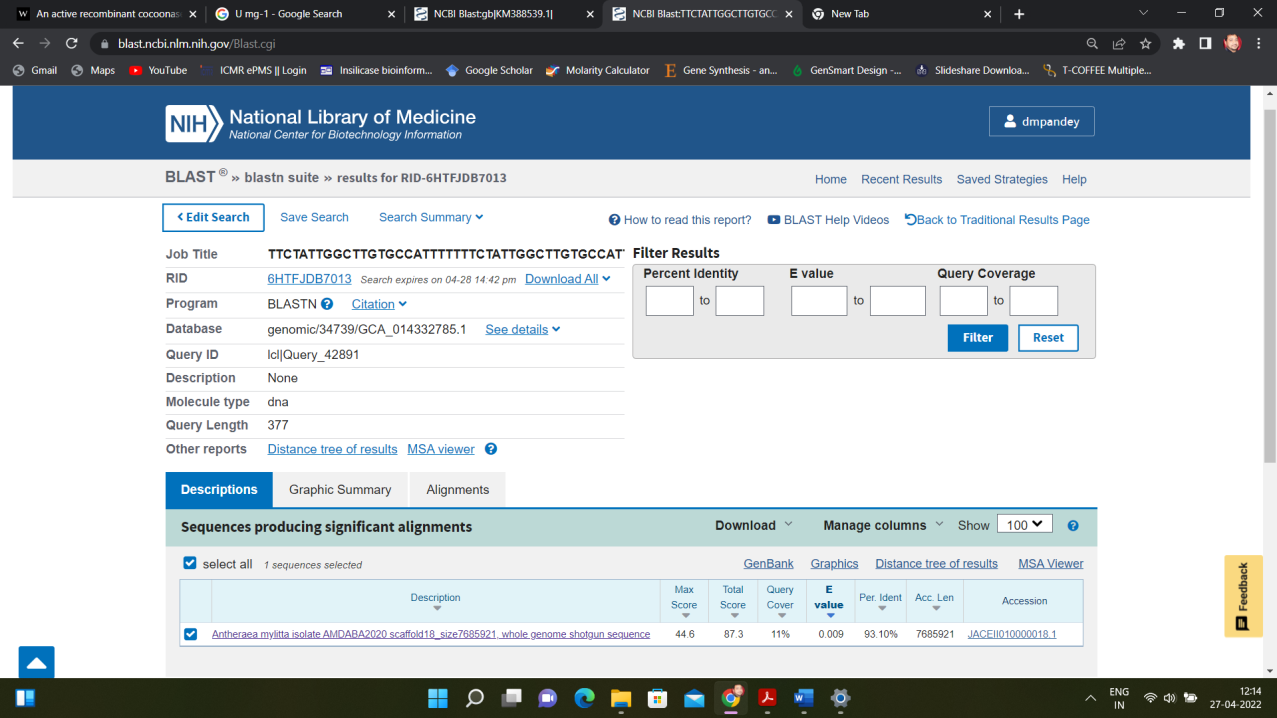


**Fig. S1.** NCBI blast result of c**ocoonase s**equence against **Antheraea mylitta - GCA_014332785.1 (AM_v1.0)]**. The blastN result shown sequences are matched with *A. mylitta* isolate AMDABA2020 scaffold18_size7685921, whole genome shotgun sequence.


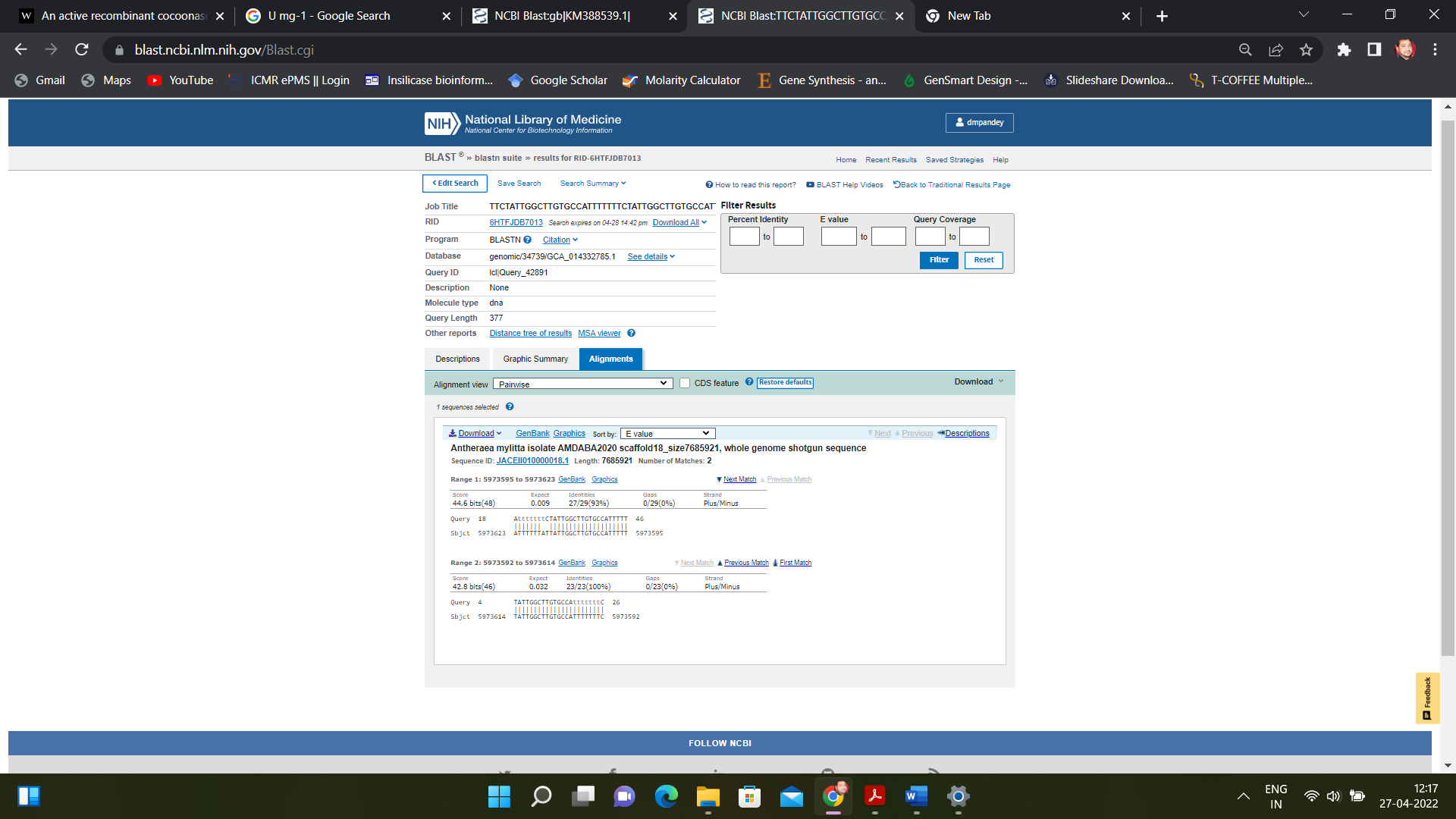


**Fig. S2.** NCBI blast result of **cocoonase s**equence against **Antheraea mylitta - GCA_014332785.1 (AM_v1.0)]**. The blast result indicated the number of 2 matches with *A. mylitta* isolate AMDABA2020 scaffold18_size7685921, whole genome shotgun sequence.

**
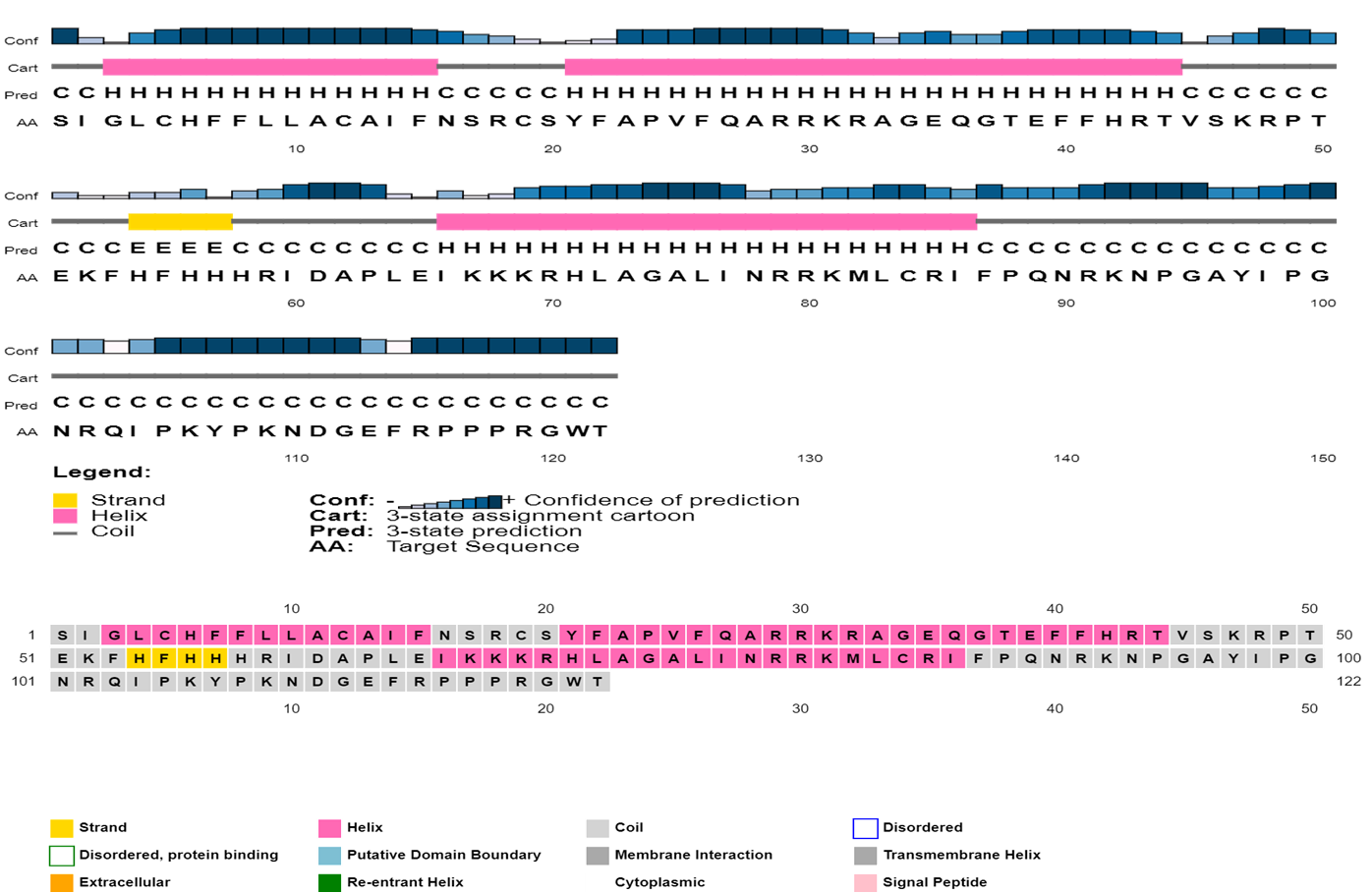
**

**Fig.** **S3.** Secondary structure prediction of *Antheraea mylitta* cocoonase (AmCoc) from PSPIRED server: **(a)** Predicted helix, strand and coil of the protein **(b)** Secondary structure map of cocoonase


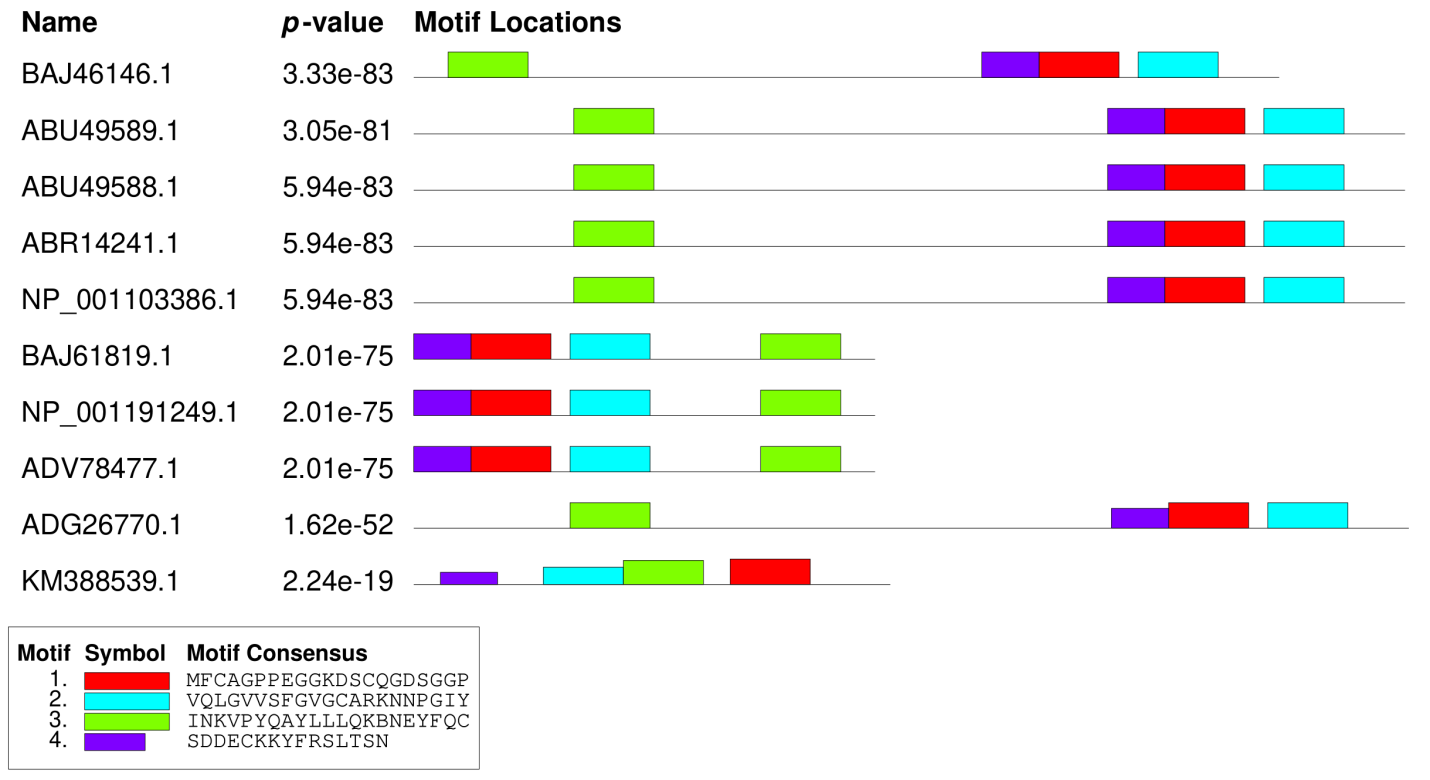


**Fig. S4.** MEME tool based result of *Antheraea mylitta* cocoonase (AmCoc) of KM388539.1 showing two strong motifs in the sequence highlighted in red (MFCAGPPEGGKDSCQGDSGGP) at position 84-104 and in lime green (INKVPYQAYLLLQKBNEYFQC) at position 56- 76


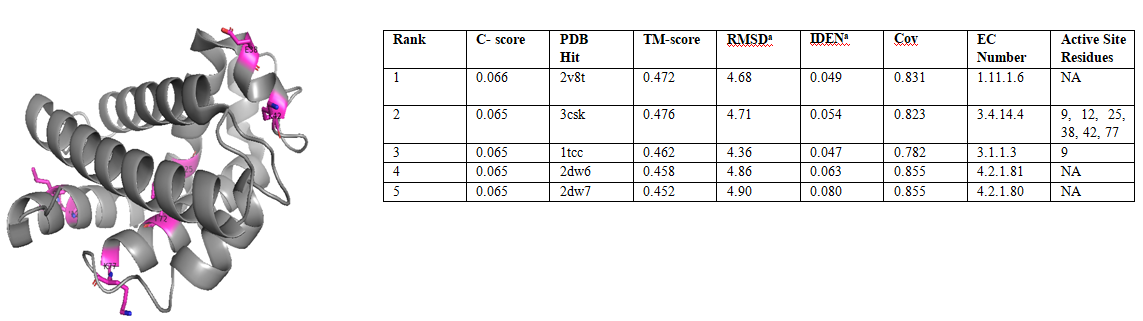


**Fig. S5.** Enzyme Commission numbers and active sites for *Antheraea mylitta* predicted cocoonase based on the template of PDB ID: 3cskA having C-score of 0.065. The predicted active-site residues are 9, 12, 25, 38, 42 and 77 is highlighted with magenta color code.
